# COVID-19 Research: Navigating the European General Data Protection Regulation

**DOI:** 10.2196/19799

**Published:** 2020-08-27

**Authors:** Regina Becker, Adrian Thorogood, Johan Ordish, Michael J.S. Beauvais

**Affiliations:** 1 ELIXIR-Luxembourg, Luxembourg Centre for Systems Biomedicine (LCSB) University of Luxembourg Esch-sur-Alzette Luxembourg; 2 Centre of Genomics and Policy McGill University Montreal, QC Canada; 3 PHG Foundation University of Cambridge Cambridge United Kingdom

**Keywords:** COVID-19, GDPR, health research, pandemic, data privacy, data protection, regulation

## Abstract

Researchers must collaborate globally to rapidly respond to the COVID-19 pandemic. In Europe, the General Data Protection Regulation (GDPR) regulates the processing of personal data, including health data of value to researchers. Even during a pandemic, research still requires a legal basis for the processing of sensitive data, additional justification for its processing, and a basis for any transfer of data outside Europe. The GDPR does provide legal grounds and derogations that can support research addressing a pandemic, if the data processing activities are proportionate to the aim pursued and accompanied by suitable safeguards. During a pandemic, a public interest basis may be more promising for research than a consent basis, given the high standards set out in the GDPR. However, the GDPR leaves many aspects of the public interest basis to be determined by individual Member States, which have not fully or uniformly made use of all options. The consequence is an inconsistent legal patchwork that displays insufficient clarity and impedes joint approaches. The COVID-19 experience provides lessons for national legislatures. Responsiveness to pandemics requires clear and harmonized laws that consider the related practical challenges and support collaborative global research in the public interest.

## Introduction

The world continues to wait expectantly for health researchers to develop effective prevention tools, tests, vaccines, and treatments for COVID-19. The collection, analysis, and timely sharing of rich health data is a key component of this unprecedented international research effort [1]. However, data protection laws are not suspended during emergencies such as a pandemic [2]. Many forms of COVID-19 health research involve the processing of personal data, including human health or genetic data. The General Data Protection Regulation (GDPR) [3] regulates the processing of personal data in the European Economic Area (EEA), which includes the 27 European Union Member States as well as Iceland, Liechtenstein, and Norway. (The United Kingdom, following the Brexit transition period, will retain the GDPR in modified form; however, these modifications have not altered the core principles of the GDPR [4,5].) In principle, the GDPR provides a toolset for the processing of personal data in a health crisis, including for health-related research [6]. One of the central reasons for adopting the GDPR and thus replacing its predecessor, the Data Protection Directive [7], was to create a harmonized data protection regime across the EEA [8]. However, a principle weakness of the GDPR is that the interpretation of multiple public health provisions and other health-related provisions is left open to national legislatures. Consequently, countries have their own solutions and requirements in many aspects where personal data—especially health and genetic data—are processed in these contexts. In particular, research institutions should be aware of the triple authorization they require under the GDPR for international data sharing: a legal basis for processing personal data, legitimation of processing special categories of data (eg, health and genetic data), and a basis for data transfers outside the EEA. Other important considerations for research institutions include respecting the rights of individual data subjects and ensuring appropriate security safeguards. The aim of our analysis is to help research institutions navigate European data protection law within the COVID-19 crisis. We also encourage European and Member State legislators to adopt a more harmonized regulatory framework that supports the needs of public health and research in a pandemic.

## A Legal Basis for Processing COVID-19 Data Under Article 6 of the GDPR

A key principle of data protection is that all personal data be processed lawfully. In other words, a research organization must have a legal basis for processing such data. The different legal bases available under the GDPR are listed in Article 6(1). The two bases most suitable for health research are the consent basis and the public interest basis. In the following, we review the strengths and drawbacks of relying on different legal bases for research in a pandemic.

### Consent (Article 6[1][a])

At first glance, consent appears to be a straightforward solution for processing personal data in COVID-19 research, as it coincides with the research ethics principle of consent. Consent under the GDPR, however, is seen to be conceptually and operationally different from the informed consent generally required by research ethics [9]. Consent under the GDPR must be freely given, specific, informed, and unambiguous, and it can be withdrawn at any time per articles 4(11) and (7). While this essentially overlaps with requirements in ethics, European data protection authorities have interpreted these four constituent elements of consent more strictly [10].

Where consent is directly sought at the time of data collection in the health care context, patients may be seen as vulnerable people, and the consent may not be valid under the GDPR because of the imbalance between the controller and the data subject [10]. Recent guidance from the European Data Protection Board (EDPB) suggests that consent to noninterventional research obtained by researchers would be legitimate as long as there was no pressure or threat of disadvantage [6]. However, the EDPB does not specify if this would be affected by the severity of illness or by the consent being obtained by the treating physician (paragraphs 21-23) [6]. Health researchers often want to analyze previously collected data as part of routine health care, particularly during a pandemic. The issue of consent regarding the secondary use of health care data for research is challenging. If the data were originally collected only for health care purposes, it would generally be necessary to re-contact the data subjects to obtain their consent to processing of their data for research purposes. Where people are in a critical status of health, which will be the case for most hospitalized patients with COVID-19, obtaining consent may not be practically possible. In addition, health care professionals are overloaded in the situation of a pandemic. It will be difficult for them to find time to provide the necessary information for consent to be valid. Staff approaching patients for consent may also increase their own risk of infection, unless innovative solutions such as electronic consent (eConsent) are implemented.

The requirement that consent be specific is another challenge of this legal basis. Broad consent to pandemic-related research is possible in principle under GDPR Recital 33, which states that “...data subjects should be allowed to give their consent to certain areas of scientific research when in keeping with recognized ethical standards for scientific research.” However, some data protection authorities may not accept such broad purposes as satisfying consent requirements without a subsequent consenting of individual projects. Indeed, the EDPB’s guidelines on consent state that when research purposes cannot be fully specified, other ways should be found to ensure the essence of consent is provided (eg, through additional consents for subsequent steps in the research [10]). In contrast, Dara Hallinan [11] argues that broad consent may still be supported in certain fields of research, such as genomics. With this position in mind, it is particularly unfortunate that the EDPB guidelines on COVID-19 and research make no mention that broader consent is suitable for pandemic research [6].

The spirit of the logic that informs data protection authorities’ insistence on additional consents for broad consent spreads into the relationship of the purpose limitation principle and processing for research. Provided that processing for research purposes is not incompatible with the initial purpose, data may be further processed for such research with appropriate safeguards (Article 5[1][b]). Where consent is the legal basis relied upon, this widening of the purpose limitation principle for research purposes is key. Without this widening, the secondary use of data for research purposes always requires that consent be obtained from the data subject. However, in case of consent, the data protection authorities may require reconsenting, as stated for example by the UK Information Commissioner’s Office [12].

Last but not least, where consent is the legal basis, the data subject retains the right to withdraw their consent at any time. Otherwise, the respect for the data subject’s decisional autonomy that finds its articulation in consent would be meaningless. If the consent that serves as the legal basis for the data processing is withdrawn, processing generally must stop and the data must be erased. Processing may only continue for other purposes based on a separate legal basis that was already established. The EDPB has pointed out on multiple occasions that a data controller cannot swap the legal basis after a withdrawal of consent [10]. This position appears to contradict GDPR Article 17(3)(d), which limits the right to erasure where this would seriously impair research or render it impossible. For ongoing research studies, deleting individual data sets may not render the research impossible, although having to repeat analyses could be cumbersome. For completed research studies, however, the situation is different, as altering the underlying database may undermine scientific reproducibility.

Given the difficulty of obtaining valid consent from patients with COVID-19 and the ambiguity around the consequences of consent withdrawal, an alternative legal basis is desirable in many situations when processing personal data for research during and after the COVID-19 crisis.

### Performance of a Task Carried Out in the Public Interest (Article 6[1][e])

As scientific research on COVID-19 aims to benefit society as a whole, using the legal basis of a task performed in the public interest appears to be a natural choice. It is also the choice suggested by the EDPB as more appropriate than consent for research in clinical trials [13] and is one of the potential legal grounds mentioned in the EDPB’s guidelines on COVID-19 and research. The availability of the public interest legal basis, however, must be established by Union or Member State law (Article 6[3]). Infectious disease or public health laws may provide the necessary legal basis as a task in the public interest. Where infectious disease legislation does not authorize a public research institution or university to process personal data for pandemic research, they may still be able to rely on their research mission given to them by law. Some countries, such as Finland [14] and Norway [15], have specified in their national legislation that the public interest legal basis may be relied upon where the processing is necessary for scientific purposes.

Overall, the performance of a recognized task in the public interest can also allow other derogations, such as those under Articles 20(3) and 21(6). As such, the public interest legal basis may allow more flexibility than other legal grounds, depending on the conditions of implementation. It is also important to realize that Article 6(1)(e), like any other legal basis outside consent, will require further national legislation implementing Article 9 for processing special categories of data (see below).

### Other Legal Grounds in Article 6

Where institutions cannot rely on public interest, legitimate interest is another option, provided that a balancing exercise is performed to ensure that the interest in processing outweighs the privacy interests of the data subject and that the fairness and transparency of the processing is demonstrated (Article 6[1][f]).

Other options apply to the health care context but do not extend to research:

1. Article 6(1)(c) supports processing to fulfill a legal obligation to which the controller is subject. This basis will cover the reporting of cases and accompanying personal data based on infectious disease acts to the relevant authorities as well as processing of data by the health authorities.
2. Article 6(1)(d) authorizes processing in the vital interest of a data subject or another natural person. Vital interest is strictly construed to mean the immediate life interests of a data subject. Relying on this article for research is too speculative: research aims to produce generalizable knowledge and ultimately to benefit public health and society; thus, it cannot be pursued for the vital interest of an individual.

Article 6(1)(d) can be a valid basis in a treatment context and could even provide an option to track people and inform them about a potential infection risk if the disease is life-threatening. In its *Opinion on the notion of legitimate interests of the data controller*, the Article 29 Working Party [16] admits that “vital interest” covers personal data processing to warn people of a potential infection during an epidemic; however, they also warn that it should not be the basis for massive collection or processing of personal data. Along the same lines, this legal basis is not even discussed in the recent statement of the EDPB on location data and contract tracing [17].

### Further Processing for Scientific Research

Recital 50 of the GDPR indicates that where further processing is compatible with the original purpose, “...no legal basis separate from that which allowed the collection of the personal data is required.” This seems to suggest that data that were not initially collected for research purposes (eg, in the health care context) may also be processed for research based on the original legal basis under which they were collected. For the compatibility of purposes, Article 5(1)(b) suggests that further processing for scientific research “...shall...not be considered to be incompatible with the initial purposes” as long as appropriate safeguards are in place (eg, those specified by Article 89[1]).

The extent to which these provisions can be applied beyond the original controller are being controversially interpreted. The European Data Protection Supervisor states in its *Preliminary Opinion on data protection and scientific research* that Article 5(1)(b) does not give a general authorization for further processing for scientific research [9]. A compatibility test should be performed, although in principle, compatibility can be assumed for both original and subsequent controllers processing data from health care for scientific research as long as appropriate safeguards are in place. By contrast, Edward Dove [18] questions if new controllers may avail themselves of the presumption of compatibility. In addition, an investigation of the Swedish government came to the conclusion that with respect to the continuation of the legal basis, only the transfer to a subsequent controller is covered by the original legal basis; the new controller must find its own valid legal ground [19]. The EDPB remains silent on the subject in their guidelines on COVID-19 and research; however, dedicated guidance on the subject of further processing is expected [9]. Given the unclear situation on the usability of the further processing exemption for collaborative research, the establishment of a legal basis based on Article 5(1)(b) in combination with Recital 50 is precarious.

## Legitimation for the Processing of Special Categories of Data (eg, Health and Genetic Data)

Personal data processed for COVID-19 research invariably includes health data. Health and genetic data are considered to be “special categories” of data. Article 9(1) of the GDPR prohibits processing of special categories of data by default. Research institutions therefore need not only a legal basis but also an additional legitimation under Article 9(2) to process health and genetic data.

Again, consent is a possible option; however, it faces many of the limitations described above. The GDPR requires that consent be both explicit and specific in the case of Article 9(2)(a). As consent for research is traditionally obtained explicitly, the larger challenge is still in the interpretation of the term “specific”. Recital 33, however, permits consent to broader areas of scientific research where purposes cannot be fully specified at the time when consent is obtained. Where processing involves special categories of data, however, the EDBP interprets this permission narrowly [10]. This may pose challenges for research on COVID-19, where a broad consent model would be necessary to cover the full range from disease mechanisms to transmission pathways to psychological or socioeconomic consequences of the disease. The information-giving duties of the data controller are increased, as is the way in which consent is recorded [10]. It is therefore useful to examine other options for legitimation.

There are two likely options for processing health and genetic data for research in the COVID-19 context. Article 9(2)(i) foresees processing for reasons of public interest in the area of public health, such as protecting against serious cross-border threats to health; therefore, this article can cover processing in the face of an epidemic. Infectious disease legislation and research into infectious diseases in an epidemic can be legitimated based on this paragraph, but only if the law provides an explicit reference to research for public health or in the event of an epidemic. Article 9(2)(j), on the other hand, covers processing that is necessary for scientific research in general, independent of the type of disease. This legitimation must also be based on Union or Member State law. Moreover, suitable and specific measures to safeguard the fundamental rights and the interests of the data subject are required. The GDPR is silent on what is meant by “suitable and specific measures to safeguard” in this context. However, these measures likely link to the requirements of proportionality, data minimization, and data security. Specific measures may include encryption, pseudonymization, minimization of sensitive data processed, training of personnel, and imposition of duties of confidentiality. The cumulative effect of these measures is to reduce the risks of processing sensitive personal data [20,21].

There is great heterogeneity among EEA countries as to whether and how they make use of these GDPR provisions. For example, regarding the implementation of Article 9(2)(j), the United Kingdom [22] and the Netherlands [23] have limited, among other conditions, the application of these provisions to research in the public interest; Sweden requires ethics approval [24]; and Finland has made defined requirements for technical safeguards [14]. The GDPR furthermore confers powers on Member States to pass additional restrictions on the processing of health and genetic data, thus heightening the potential for divergence (Article 9[4]). Some countries have even adopted separate rules for health data on one hand and for genetic data on the other. For example, Ireland has reintroduced explicit consent as a prerequisite (without a specific government declaration in narrow circumstances) [25]. Where health care data are used, professional secrecy rules must also be considered. These conditions follow national or regional health care legislation.

In consequence, a confusing patchwork of heterogeneous provisions for processing personal health and genetic data for pandemic research has been created across Europe. This inharmonious assortment of differing solutions is not helpful where global cross-border sharing and timely solutions are needed. This is also demonstrated by the EDPB’s guidelines on COVID-19 and research [6]. The recommendations remain on a generic level and refer to Member State solutions without further discussing the context and requirements of the pandemic. For a problem that Europe confronts in unison, we are required to return to the Member State level to find solutions.

## Personal Data Transfer Outside the EEA

In the face of a truly global pandemic, there is a clear need for researchers to collaborate and rapidly share data internationally. Chapter V of the GDPR imposes limitations on the transfer of personal data outside the EEA, aiming to ensure that Europeans’ personal data are subject to essentially equivalent levels of protection when sent to other countries [26].

Among the number of instruments that can be used in the context of a pandemic, an adequacy decision granted by the European Commission is the most straightforward, as it allows data exchange under the same conditions as within the EEA (Article 45). The utility of this instrument remains limited, however, as only thirteen jurisdictions have received recognition [27]. Adequacy decisions require careful study, as they sometimes cover only certain sectors. Moreover, the continued validity of adequacy decisions may be imperiled if recipient third countries adopt aggressive data collection and processing practices in response to COVID-19 [28,29].

Another option is to adopt one of the appropriate safeguards for transfer is introduced in Article 46. Legally binding and enforceable instruments between public authorities and bodies may be a satisfactory safeguard, particularly for the exchange between health authorities (Article 46[2][a]). For public research, however, few enforceable instruments currently exist. Institutions may also rely on the standard contractual clauses provided by the European Commission (Article 46[2][c]). A notable problem with these clauses, however, is that US government departments, public universities, and academic health centers cannot consent to dispute resolution in European courts [30]. Alternative contractual clauses or administrative arrangements between public authorities and bodies will require approval by the data protection authority and are not likely to provide ad hoc or fast solutions (Article 46[3][a]).

The pandemic, however, may justify the reliance on derogations for specific situations. Cross-border transfers can be legitimated by explicit consent under Article 49(1)(a); however, the same practical barriers must be overcome. For example, the data subject must be informed about the particular transfers envisaged; thus, it is not possible for a data subject to give blanket consent to any future, unspecified transfers outside the EEA [31]. The EDPB furthermore notes that explicit consent is only suitable for certain situations, such as for private entities conducting COVID-19 research; however, it provides no further guidance other than its guidelines on COVID-19 and research (paragraph 67) [6]. Transfers necessary for important reasons of public interest, where this public interest is recognized in Union law or national law (Articles 49[1][d] and 49[4]), could provide a more appropriate solution in the acute situation of a pandemic, in particular because cross-border research collaboration is an important aspect in fighting a pandemic. However, the EDPB also cautions that transfers according to this derogation shall “not become the rule in practice,” be restricted to “specific situations,” and be “strictly necessary” for the purposes for processing [31]. In the specific situation of the COVID-19 outbreak, the EDPB has conceded “that the fight against COVID-19 has been recognized by the EU and most of its Member States as an important public interest,” grounding this claim in provisions of EU law interpreted through the prism of national measures by Member States adopted in response to the crisis (paragraphs 62-67) [6]. Time will tell if grounding international transfers in the public interest will be an accepted solution across Europe.

Where none of these options can be applied, researchers who are not acting within a public authority in the exercise of its mandate may at least claim as an immediate measure that the transfer is necessary for purposes of compelling legitimate interest that are not overridden by the interests or rights and freedoms of the data subject (Article 49[1]). Such transfers are subject to myriad restrictions; for instance, the transfer must not be repetitive, only concern a limited number of data subjects, implement suitable safeguards, and be underpinned by proof to the relevant data protection authority that no other option is available (Article 49[1]). This is, at best, a stopgap solution.

The EDPB does recognize the importance of international transfers for pandemic research, albeit perhaps half-heartedly (transfers are “probably” required) [6]. The EDPB also suggests that the existing provisions under the GDPR are sufficient to conduct such transfers. Given that the European research community is still waiting for solutions for international research data sharing to be developed outside the pandemic context, the lack of a clear route remains a serious concern.

## Derogation of Data Subject Rights

An additional hurdle is created through the strict information-providing obligations research institutions have toward data subjects under the GDPR. Extensive information on the use of data must be provided to the data subject before collection, including information about the legal basis for processing and the intention to transfer personal data to third countries (Article 13). There are only narrow exceptions to the requirement that direct information be given to the data subject. Exceptions are foreseen in cases where data are not obtained directly from the data subject (Article 14). COVID-19 research involving secondary use of data collected during the care of seriously ill patients may fall under this exception. Where informing the subject of such research would be impossible or would involve disproportionate effort, general information may alternatively be shared on the controller’s web page or in public announcements. The research must be subject to the appropriate safeguards described in Article 89. However, even for the case of secondary use of data collected in health care, the original controller (ie, the health care provider) would still be obligated to inform the data subject about the data sharing for further processing in the research context.

The critiques about the implementation of these requirements are the same as above: providing additional information in the clinical context during an emergency, with many patients in a serious state of illness and experiencing breathing problems, may not be feasible. As no derogation is foreseen, it is not clear how the data protection authorities will judge any retrospective information provided to the patients who survive their disease. A way out could be based on provisions allowing the restriction of all data subjects’ rights in matters of important public interest, such as public health (Article 23). Again, national or European laws must provide the necessary framework. Only a few Member States have implemented such derogations in their national data protection legislation; many of these are superficial, stating the possibility of derogations for public health, among others [23,32]. No detailed provisions have been made specific to related research aspects for the preservation of public health. The resulting legal uncertainty hampers responsive research during a pandemic if appropriate information cannot be provided before starting the research.

## Regulations in a State of Emergency

Many countries in the EEA provide for the possibility that in emergency situations, the government can enact laws to cope with a crisis such as a pandemic [33-36]. These provisions can restrict the rights of citizens, including their data protection rights, as foreseen in the GDPR itself (Article 23). In a pandemic, such regulations can derogate from data subjects’ rights and provide a legal basis for processing beyond the existing legal framework. These emergency powers do not amount to *carte blanche*; the measures must be necessary, appropriate, and proportionate to the aim pursued. The EDPB also points out that measures implemented based on emergency situations should be strictly limited to the duration of the emergency [37]. Moreover, to plausibly rely on such powers for research, there must be a close, proximate connection between the research conducted and the pandemic response. Such measures are also subject to oversight by national constitutional or administrative courts, as well as the Court of Justice of the European Union and the European Court of Human Rights.

Debates around emergency derogations in the context of data processing have thus far focused largely on location tracking or contact tracing of people through mobile phones rather than on health research. Such measures can be used to identify people at risk and to monitor adherence to social distancing. The EDPB has issued a letter to the European Commission stating that an enactment of national laws would be a good way to provide a solid legal framework to define the scope and also the limited duration of the use of mobile phone information, disabling of tracking systems, and deleting of data once the crisis is over [38]. At the same time, the EDPB insists that the use of an app should be voluntary and not made a mandatory measure or involve any disadvantages for those not opting into the use of the app [38]. The rights of the data subjects should not be compromised but should rather be upheld to retain the trust and buy-in of the citizens. Along these lines, a joint statement has been signed by more than 550 researchers worldwide calling for privacy preserving technologies, strict limitation to COVID-19 purposes, and a voluntary basis for the use of any contact tracing app [39].

Our discussion below brings attention to the need for similar emergency derogations for health research during the pandemic. The two debates are connected: information collected as part of tracking and contact tracing could support research into the course of the pandemic and the mechanisms of virus transmission.

An example of emergency legislation with specific provisions for research was introduced in Italy, where the obligation for prior consultation of the data protection authority where no consent can be obtained can be waived under certain circumstances [40]. This requirement is arguably disproportionate even in normal circumstances; however, even in the present state of the COVID-19 crisis, it is only applicable to clinical trials, observational medicinal product studies, and compassionate therapeutic use that is essential for combating the disease as well as for COVID-19 research projects of the Institutes of Scientific Hospitalization and Treatment funded by the Ministry of Health. Also, derogations from GDPR Article 13 have been made based on Article 23(1)(e). The Italian waiver demonstrates how the current pandemic exposes weaknesses in data protection legislation for research and how emergency regulations can be used as a quick, albeit temporary, remedy.

## Additional Obligations Under Data Protection Law

The general obligations of controllers to process personal data apply to pandemic research. Article 5 comprises the data protection principles of lawfulness, fairness, transparency, purpose limitation, data minimization, accuracy, storage limitation, integrity, and confidentiality, as well as accountability of the controller. Some of these aspects have been discussed above. The implementation of data protection by design and default is largely described in Chapter IV of the GDPR. Technical and organizational measures must be taken to ensure that the rights and freedoms of data subjects are not unduly compromised. Before they process special categories of data in the COVID-19 context, research institutions will most likely need to perform data protection impact assessments (DPIAs), which act as both a safeguard and a mechanism for enhancing transparency and accountability. Researchers can find guidance regarding the specific rules in their country from dedicated lists of processing requiring a DPIA on the webpage of their data protection authority. A DPIA will need to include further documentation on the processing and the safeguards introduced as well as a subsequent risk analysis covering the potential impact on the rights and freedoms of the data subjects. The French supervisory authority provides a useful guide and associated tool in English and other European languages [41].

In any case, the GDPR requires that security safeguards be proportionate to the risks of processing (Article 32). Research institutions must therefore adopt stricter security safeguards for processing sensitive health and genetic data. Some countries have introduced specific legislative provisions for security safeguards that the research institutions need to be aware of, including technical measures when processing personal data for scientific research (eg, Luxembourg [42]) and when processing health or genetic data (eg, Ireland [25]). The requested safeguards differ between countries.

## Conclusion

The GDPR has foreseen mechanisms that enable research in a pandemic, which include processing sensitive data of vulnerable people, the need for fast action, and global data sharing. However, these mechanisms depend largely on national implementation. Our analysis demonstrates that variation across national implementations hampers a coordinated global research response in the fight against COVID-19. This is also reflected by the request for a mandate by the EDPB to develop guidelines on short notice [43]. The subsequently published guidelines on COVID-19 and research [6] contain only limited advice that takes into account specific challenges in the research contexts (eg, hospitalized people who are unable to give consent, challenges in information-giving obligations). They largely refer to the principal framework and the possibility of measures based on Member State implementation of the GDPR, which remain incomplete and heterogeneous. Research institutions must investigate their scope of action and requirements on a national level and, when in doubt, contact the competent data protection authorities. Without legal clarity from European and national legislators, certain research institutions may need to lead the response based on their best interpretations of current laws.

As lessons learned from this crisis, the EEA countries should review and adapt their legislation, making particular use of enabling articles such as those relating to processing health data for reasons of the public interest in public health (Article 9[2][i]) and derogations to data subject rights and controller obligations based on public interest (Article 23). To date, few countries have used these for the benefit of research to contribute to public health. This indicates that many legislators and data protection authorities still lack deep understanding of the needs of health research and the reason for the privileged status of scientific research in the GDPR. Learning from the COVID-19 crisis, they should develop an operational framework that is appropriate to the needs of global research in a pandemic and provides legal certainty for researchers to act upon. In establishing national frameworks, legal interoperability should be a key consideration during the legislative process to allow all researchers to participate in joint research efforts under compatible conditions and to efficiently set up and manage cross-border cohorts. The EDPB could act as a coordinator or convener of such a process. This will allow researchers to combat not only future pandemics but also other pressing public health priorities.

## Abbreviations

DPIA: data protection impact assessment
eConsent: electronic consent
EDPB: European Data Protection Board
EEA: European Economic Area
GDPR: General Data Protection Regulation
